# ^18^O-assisted dynamic metabolomics for individualized diagnostics and treatment of human diseases

**DOI:** 10.3325/cmj.2012.53.529

**Published:** 2012-12

**Authors:** Emirhan Nemutlu, Song Zhang, Nenad O. Juranic, Andre Terzic, Slobodan Macura, Petras Dzeja

**Affiliations:** 1 Division of Cardiovascular Diseases, Department of Medicine, Mayo Clinic, Rochester, Minn, USA; 2 Department of Biochemistry and Molecular Biology, College of Medicine, Mayo Clinic, Rochester, Minn, USA; 3 Department of Analytical Chemistry, Faculty of Pharmacy, University of Hacettepe, Ankara, Turkey

## Abstract

Technological innovations and translation of basic discoveries to clinical practice drive advances in medicine. Today's innovative technologies enable comprehensive screening of the genome, transcriptome, proteome, and metabolome. The detailed knowledge, converged in the integrated “omics” (genomics, transcriptomics, proteomics, and metabolomics), holds an immense potential for understanding mechanism of diseases, facilitating their early diagnostics, selecting personalized therapeutic strategies, and assessing their effectiveness. Metabolomics is the newest “omics” approach aimed to analyze large metabolite pools. The next generation of metabolomic screening requires technologies for high throughput and robust monitoring of metabolite levels and their fluxes. In this regard, stable isotope ^18^O-based metabolite tagging technology expands quantitative measurements of metabolite levels and turnover rates to all metabolites that include water as a reactant, most notably phosphometabolites. The obtained profiles and turnover rates are sensitive indicators of energy and metabolic imbalances like the ones created by genetic deficiencies, myocardial ischemia, heart failure, neurodegenerative disorders, etc. Here we describe and discuss briefly the potential use of dynamic phosphometabolomic platform for disease diagnostics currently under development at Mayo Clinic.

## Integrated “omics” approach

Living cells represent an integrated and interacting network of genes, transcripts, proteins, small signaling molecules, and metabolites that define cellular phenotype and function. Traditionally the focus of biomedical research was on individual genes, single protein targets, single metabolites, and metabolic or signaling pathways. This “molecular reductionist” paradigm was based on the assumption that identifying genetic variations and molecular components would lead to discovery of cures for human diseases. However, most of diseases are complex and multi-factorial and the disease phenotype is determined by the alterations of multiple genes, pathways, proteins and metabolites (at cellular, tissue, and organismal levels). Therefore, an integrated “omics” approach is more viable direction for uncovering alterations in metabolic networks, disease mechanisms, and mechanisms of drug effects.

**Figure 1:**
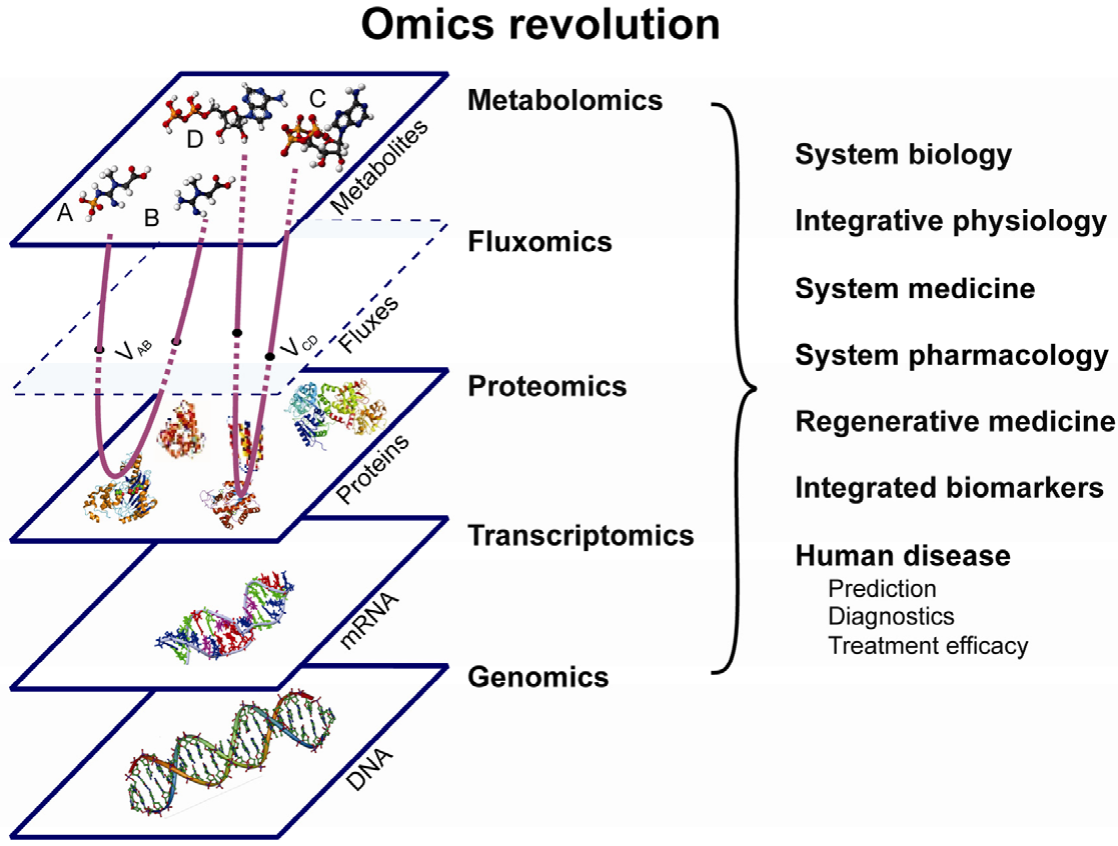
The “omics revolution” – an integrated comprehensive “omics” approach combining genomics, transcriptomics, proteomics, metabolomics, and fluxomics for advancement of systemic sciences and for human disease diagnostics and treatment. After Nielsen and Oliver (1).

Recent advent of large-scale metabolomics and fluxomic (metabolite dynamics and metabolic flux analysis) completed the “omics revolution” (Figure 1), where genomics, transcriptomics, proteomics, metabolomics, and fluxomics all together complement phenotype determination of living organism. Such integrated “omics” cascades provide a framework for advances in system and network biology, integrative physiology, and system medicine as well as system pharmacology and regenerative medicine. Noteworthy is the “reverse omic” approach or “metabolomics-informed pharmacogenomics,” where discovery of specific metabolite changes have led to discovery of genetic alterations (2). Therefore, bringing new “omics” technologies to clinical practice will improve disease diagnostics and treatment by targeting drugs and procedures for each unique transcriptomic and metabolomic profiles.

## Dynamic metabolomic technologies

Comprehensive characterization of metabolic networks and their function requires quantitative knowledge of metabolite concentrations (metabolomics) and metabolite fluxes (fluxomics). Analytically, this could be perceived as a determination of concentrations and turnover rates of a large number of small molecules (metabolites) from tissue or body fluids. Thus, metabolomics and fluxomics methods heavily rely on the information-rich analytical techniques, most notably nuclear magnetic resonance (NMR) spectroscopy and mass spectrometry. Stable isotope ^18^O-assisted ^31^P NMR and mass spectrometry provide means for simultaneous measurements of phosphorous-containing metabolite (phosphometabolite) levels and their respective turnover rates in tissue and blood samples. The ^18^O labeling of metabolites is based on the incorporation of the ^18^O nuclei (from H_2_^18^O), into phosphate group with each act of adenosine triphosphate (ATP) hydrolysis, and subsequent distribution of ^18^O-labeled phosphoryls among phosphate-carrying molecules. ^18^O is a natural and stable isotope of oxygen. When tissue or cells are exposed to media containing water with a known percentage of ^18^O, H_2_^18^O rapidly equilibrates with the cellular water, and ^18^O from water is incorporated into cellular phosphate metabolites proportionally to the rate of the involved enzymatic reactions (3). All major phosphometabolites and their turnover rates can be quantified using ^18^O-assisted ^31^P NMR spectroscopy and much faster by the use of mass spectrometry. The ^18^O isotope effect is readily visible as ^18^O induced ^31^P chemical shift in high-resolution ^31^P NMR spectra, and as 2 amu change of mass in mass spectra. Thus, stable isotope tracer-based metabolomic technologies allow simultaneous quantitative determination of metabolite levels and their turnover rates with subsequent evaluation of metabolic network dynamics.

Dynamic phosphmetabolomic platform under development at Mayo Clinic (Rochester, MN, USA) is shown in Table 1. This analytical platform includes metabolome determination, providing static metabolic profile, and dynamic phosphometabolomics where phosphometabolite turnover rates, metabolic flux distribution in phosphotransfer networks, and dynamics of metabolic cycles are analyzed. Based on the data obtained by these technologies, a correlation matrix and predictive algorithm is being developed for disease diagnostics.

**Table 1:** Dynamic phosphmetabolomic platform*

Metabolomics →	Dynamic phosphometabolomics (stable isotope ^18^O-labeling) →	Clinical metabolomics and ^18^O phosphometabolomics
Metabolome, static metabolic profile. *Technologies:* Tissue, cell and body fluid samples GC/MS LC/MS ^1^H NMR ^31^P NMR	Phosphometabolite turnover rates, metabolic flux distribution analysis in phosphotransfer networks and dynamics of metabolic cycles. *Technologies:* ^18^O-labeling of tissue, cell and whole blood samples ^18^O-assisted GC/MS ^18^O-assisted LC/MS ^18^O-assisted ^31^P NMR Model-based flux analysis	Metabolomics + Dynamic phosphometabolomics = system and network approach, disease mechanisms, more accurate disease prediction, diagnosis, treatment choices and efficacy. *Technologies:* ^18^O-labeling of tissue, cell and whole blood samples ^18^O-assisted GC/MS ^18^O-assisted LC/MS Correlation and predictive algorithms

*Abbreviations: GC – gas chromatography; MS – mass spectrometry; LC – liquid chromatography; NMR – nuclear magnetic resonance.

## Dynamic metabolomic profiling

The analysis of metabolic fingerprints left by disease processes and metabolic monitoring of disease progression or treatment efficacy plays a crucial role in personalized and predictive medicine. Metabolomic profiling may not be enough to predict the phenotype as it gives only an instant static picture of the physiology of a living organism. To entirely understand metabolic phenotypes and network dynamics, quantitative knowledge of metabolite turnover rates is required (4-13). This is because significant alterations in metabolic flux could take place without obvious changes in metabolite concentrations, especially metabolites associated with high flux/turnover rates (6,14). Stable isotope tracer-based metabolomic technologies allow for simultaneous determination of metabolite levels and their turnover rates with subsequent evaluation of metabolic network dynamics (10,11,13-16). For example, ^13^C labeling is widely used to track turnover of the carbon backbone of metabolites and label propagation through metabolic networks (17-19). However, it does not provide data on the status of the phosphate-containing metabolite based cellular energetic. On the other hand, ^18^O isotopes are conveniently used to follow phosphate transfer rates of energetically and signal transduction important biomolecules (3,10,16,20-24). As water is involved in many enzymatic reactions, ^18^O from the H_2_^18^O can be incorporated into array of metabolites. This expands the use of ^18^O technology to labeling of many non-phosphate containing metabolites, which in turn enables observing of large metabolic networks in intact tissues and alterations in human disease. In this regard, ^18^O stable isotope labeling is widely used in quantitative proteomics to detect changes in protein levels and in their turnover rates (25,26). We believe that the analysis of phosphometabolite turnover rates using ^18^O labeling (in whole fresh blood, blood cell fractions, plasma, and tissue samples) will soon become practical enough to be routinely used in translational research, metabolic phenotyping, biomarker development, and ultimately in diagnostics and treatment of human diseases.

## Dynamic phosphometabolomics

Phosphate is indispensable to life activity and is the most common fragment in terms of the frequency of occurrence in the metabolome of living organisms (27,28). Phosphometabolite dynamics can be a predictor of atrial fibrillation and prothrombotic events critical in prevention of stroke and myocardial infarction (29). ^18^O-assisted gas chromatography/mass spectrometry (GC/MS), liquid chromatography/mass spectrometry (LC/MS), and ^31^P NMR technologies fill a critical gap in metabolomics technologies by providing a currently unavailable tool for analysis of perturbations in cellular energetic and metabolic signaling networks induced by diseases or genetic and acquired metabolic deficiencies (29). It can be further developed to clinically-useful stable-isotope based bioanalytical platform for analysis of phosphometabolite levels and turnover rates with high precision and extended capacity for molecular recognition and automated isotopomer analysis (30,31).

Phosphometabolomics is a new emerging area in metabolomic analyses targeting over 400 phosphometabolites critical in energetic and signaling processes. Currently, no single analytical tool fulfills all requirements for an ideal phosphometabolomic profiling, due to the physicochemical diversity of phosphometabolites, from hydrophobic phospholipids to hydrophilic phosphocarbohydrates, and phosphoamino- and non-phosphoamino-organic acids. Thus, different analytical techniques must be used to generate a comprehensive metabolomics profile (7,32,33). We established a dynamic phosphometabolomic platform that includes ^18^O-assisted GC/MS, ^18^O-assisted ^31^P NMR, together with ^1^H NMR and high pressure liquid chromatography (HPLC) (29). ^18^O-assisted GC/MS technology allows separation and quantitation of ^18^O/^16^O isotope ratios in phosphoryl metabolites with a molecular mass <500 Da. ^18^O-labeling of higher molecular weight phosphates and oligophosphates, such as ATP, can be analyzed by LC/MS and ^31^P NMR. 2D ^1^H-^31^P NMR total correlation spectroscopy (TOCSY) and 1D ^1^H NMR and HPLC complement the analysis, identification, and fractionation of phosphometabolites.

The understanding of the dynamics of oligophosphates at different phosphoryl moieties provides unique knowledge of energetic, signal transduction, and biosynthetic processes in the cell (3,16,20-24,34). For instance, all ^18^O isotopologues in ATP can be directly translated into activity of adenylate kinase, ATP synthase, ATPase, pyrophosphokinase, nucleotidyl transferases, and other energetic enzymes (16,31,34-37). We start to develop software for automatic analysis of isotope distribution which will enable monitoring of phosphometabolite turnover rates in whole fresh blood and tissue samples in minutes. Blood plasma and cells carry a wealth of metabolic information about health status and disease biomarkers. Blood phosphometabolite and nucleotide dynamics are important in regulation of blood flow and coagulation and their alterations may precipitate the risk of thrombosis and hypertension.

Stable isotope ^18^O labeling is a suitable technique to follow cellular phosphate group dynamics through phosphotransfer network. Thus, the ^18^O-based metabolite tagging technology bridges traditional static metabolomics with fluxomics allowing dynamic analysis of metabolic networks. For example, by using ^18^O-assisted analytical platforms, we were able to determine dynamic metabolic signatures of pacing-induced heart failure and metabolic phenotypes of transgenic mouse models of human diseases, associated with K-ATP channels and phosphotransfer enzyme deficiencies, as well as the effects of environmental stress (such as hypoxia) on global changes in energetics and metabolic signaling networks (9,15,38-43).

A large scale ^18^O stable isotope-based metabolomic technology enables determination of dynamic metabolomic signatures critical in understanding mechanisms of metabolic deficiencies and for early diagnosis and monitoring of human diseases. An advanced large scale clinically-usable phosphometabolomic technology for accurate monitoring of phosphometabolite turnover rates in human tissue, plasma and whole fresh blood samples would improve disease risk assessment, diagnosis, prognosis, and treatment of body energy and metabolic imbalance diseases. Collectively, a novel stable isotope methodology (^18^O-based mass spectrometry and ^31^P NMR spectroscopy) will bring to clinical practice advanced stable isotope-based metabolomic profiling for risk stratification, prediction of disease course, and personalized treatment of diseases by targeting drugs and procedures for each individual metabolomic profile.
